# Thermally Degradable Poly(*n*-butyl acrylate) Model Networks Prepared by PhotoATRP and Radical Trap-Assisted Atom Transfer Radical Coupling

**DOI:** 10.3390/polym14040713

**Published:** 2022-02-12

**Authors:** Michael R. Martinez, Ziye Zhuang, Megan Treichel, Julia Cuthbert, Mingkang Sun, Joanna Pietrasik, Krzysztof Matyjaszewski

**Affiliations:** 1 Department of Chemistry, Carnegie Mellon University, 4400 Fifth Avenue, Pittsburgh, PA 15213, USA; mrmartin@andrew.cmu.edu (M.R.M.); ziyez@andrew.cmu.edu (Z.Z.); mtreiche@andrew.cmu.edu (M.T.); jcuthber@andrew.cmu.edu (J.C.); mingkans@andrew.cmu.edu (M.S.); 2 Faculty of Chemistry, Institute of Polymer and Dye Technology, Lodz University of Technology, Stefanowskiego 16, 90-537 Lodz, Poland; joanna.pietrasik@p.lodz.pl

**Keywords:** ATRP, ATRC, network, degradable, polyacrylate, nitroxide

## Abstract

Model poly(*n*-butyl acrylate) (PBA) networks were prepared by photoinduced atom transfer radical polymerization (photoATRP), followed by curing of polymer stars via atom transfer radical coupling (ATRC) with a nitrosobenzene radical trap. The resulting nitroxyl radical installed thermally labile alkoxyamine functional groups at the junctions of the network. The alkoxyamine crosslinks of the network were degraded back to star-like products upon exposure to temperatures above 135 °C. Characterization of the degraded products via gel permeation chromatography (GPC) confirmed the inversion of polymer topology after thermal treatment.

## 1. Introduction

Crosslinked polymer networks (commonly referred to as gels, elastomers, or thermosets) are versatile materials with mechanical properties tunable by both chemical composition and topology [1]. Thus, polymer networks have been successfully used as soft elastomers [2,3,4,5], membranes [6,7], porous materials [8,9], pH responsive gels [10], and medical devices [11]. Networks can be self-healing or degradable if dynamic or labile bonds are installed at the junctions [12,13,14,15,16,17]. The bonds can be used to alter polymer topology via post-polymerization modification upon exposure to chemical or external stimuli. The topology of a polymer network is also influenced by the curing conditions. For example, networks prepared by (free) radical polymerization, reversible addition fragmentation chain transfer (RAFT), and atom transfer radical polymerization (ATRP) demonstrated subtle differences in swelling ratios and rheology due to nuances in crosslinking chemistry, despite an identical chemical composition [12]. 

Networks can be prepared by copolymerization of monomers with multi-functional crosslinkers, or by coupling end-linked multifunctional polymers in the synthesis of “model networks”. Model networks have uniform crosslink density and molecular weight between entanglements because the distance between junctions is closely related to the topology of the starting materials. The curing reaction is often highly favorable and achievable using functional groups which could be easily attached to a polymer end-group. Model networks have been cured by condensation [18], hydrosilylation [19], azide-alkyne cycloadditions [20,21], thiol-ene reactions [22], Diels–Alder cycloadditions [23], and radical trapping using multifunctional crosslinkers with (2,2,6,6-tetramethylpiperidin-1-yl)oxyl (TEMPO) moieties [24]. 

**Scheme 1:**
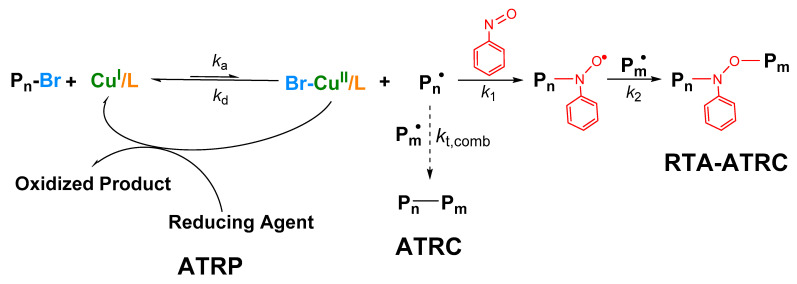
Left: Atom transfer radical polymerization (ATRP) with regeneration of activators. Center: Atom transfer radical coupling (ATRC) utilizes a macroinitiator bimolecular termination via combination to couple polymers. Right: Radical trap-assisted ATRC (RTA-ATRC) is accomplished by trapping a polymeric radical with a nitroso compound to yield a nitroxide-capped polymer, which can trap a second polymeric radical to yield a coupled polymer with the alkoxyamine functionality at its core.

Atom transfer radical coupling (ATRC) has not yet been used to prepare model networks despite its synthetic simplicity and high selectivity to halogen chain-ends. An ATRC process relies on the ATRP equilibria between a polymer macroinitiator with halogen chain-end functionality (CEF) and the reduced Mt^n^/L transition metal catalyst with the chain-end radical and oxidized X-Mt^n + 1^/L catalyst [25]. Coupling is accomplished by bimolecular termination via combination between two polymeric radicals, leading to accumulation of the oxidized X-Mt^n + 1^/L catalyst unless reducing agents such as tin (II) ethyl hexanoate, ascorbic acid, or zero-valent metals are used to (re)generate the reduced Mt^n^/L catalyst (Scheme 1) [26,27]. ATRC was previously used to prepare high molecular weight polymers, polymer macrocycles, and branched polymers [26,27,28,29]. 

A radical trap can be used to install an alkoxyamine bond during the coupling reaction via the radical trap-assisted atom transfer radical coupling (RTA-ATRC) approach (Scheme 1). In RTA-ATRC, the polymer macroinitiator prepared by ATRP is activated by the ATRP catalyst to yield a chain-end radical which is trapped by a TEMPO radical trap [30,31] or nitroso compound (i.e., nitrosobenzene) [32,33,34,35,36,37,38,39]. The latter reaction yields a polymer with terminal nitroxide CEF capable of trapping a second macroinitiator to yield a coupled polymer with an alkoxyamine functional group at the center. This approach was also used to prepare high molecular weight linear polymers, branched polymers, block copolymers, and cyclic copolymers [32,33,34,35,36,37,38,39]. 

Alkoxyamines are thermally unstable and can decompose above a temperature of ~120 °C. Networks crosslinked via radical trapping with TEMPO showed negligible creep at ambient temperatures due to the high activation energy of alkoxyamine activation, but dynamic-covalent behavior was observed at elevated temperatures once alkoxyamine exchange became favorable [24,40,41,42]. Thus, alkoxyamine-crosslinked networks could be thermally decomposed back to the linear polymer precursors by heating the network in stoichiometric excess of monofunctional TEMPO [24]. 

In this manuscript, thermally degradable poly(*n*-butyl acrylate) model networks were prepared by RTA-ATRC of polymer stars with a nitrosobenzene radical trap. The degradation of the network is characterized in detail in the solid state through dynamic mechanical analysis (DMA) at variable temperatures, and in solution by gel permeation chromatography (GPC). 

## 2. Materials and Methods

### 2.1. Chemicals

*n*-Butyl acrylate (BA, 99%, Aldrich, St. Louis, MO, USA) was passed through basic alumina column to remove radical inhibitors prior to use. Anisole (Aldrich, 99%), nitrosobenzene (NBz, 99%, Aldrich, St. Louis, MO, USA), α-bromoisobutyryl bromide (BiBBr, 98%, Aldrich, St. Louis, MO, USA), pentaerythritol (for synthesis Aldrich, St. Louis, MO, USA), tin (II) ethylhexanoate (Sn(EH)_2_, 98%, Aldrich, St. Louis, MO, USA), tetrahydrofuran (THF, ACS grade, Fisher Scientific, Hampton, NH, USA), copper(II) bromide (CuBr_2_, 99%, Aldrich, St. Louis, MO, USA), *N*,*N*-dimethylformamide (DMF, ACS grade, Fisher Scientific, Hampton, NH, USA), deuterated chloroform (CDCl_3_, 99.8%, Cambridge Isotope Laboratories, Tewksbury, MA, USA), and tris [2-(dimethylamino)ethyl]amine (Me_6_TREN, Aldrich, St. Louis, MO, USA) were used as received. 

### 2.2. Instrumentation

#### 2.2.1. Gel Permeation Chromatography (GPC)

GPC was used to characterize the molecular weight and dispersity of soluble polymer samples with THF as the eluent. The GPC set-up consisted of a Waters 515 HPLC pump (Waters, Milford, MA, USA), Waters 2414 refractive index detector (Waters, Milford, MA, USA), and PSS columns (SDV 10^2^, 10^3^, 10^5^ Å) (PSS, Philadelphia, PA, USA). The GPC utilized THF as the eluent at a flow rate of 1 mL min^−1^ at 35 °C. Linear PMMA standards were used for GPC calibration and a toluene internal standard was used as the flow marker. The molecular weight and dispersity measurements were analyzed with WinGPC 7.0 software (PSS, Philadelphia, PA, USA). 

#### 2.2.2. Nuclear Magnetic Resonance (^1^H NMR)

^1^H NMR was carried out with Bruker Ultrashield 500 MHz NMR (Bruker, Billerica, MA, USA) with CDCl_3_ as the solvent. The chemical shift of the materials was measured relative to the CHCl_3_ protons as the internal standard. Bruker Topspin (v. 4.0.7) software was used for data processing (Bruker, Billerica, MA, USA). 

#### 2.2.3. Thermogravimetric Analysis (TGA)

TGA was performed on a TA Instruments 550 TGA (TA Instruments, New Castle, DE, USA) under an air atmosphere with a heating rate of 10 °C/min. The tests utilized ~15 mg of polymer samples loaded into a platinum sample tray. 

#### 2.2.4. Dynamic Mechanical Analysis (DMA)

Mechanical properties of the polymer gels were assessed in the dry state using an Anton Paar MCR-302 Rheometer (Anton Paar, Graz, Austria) fitted with a 25 mm diameter stainless-steel parallel plate tool. The gels were characterized using disk-shaped samples with a thickness of 1–2 mm and a diameter (D) of ~12 mm. The poly(*n*-butyl acrylate) polymer stars and degraded networks were cast as films with a thickness of ~1 mm from THF directly onto the stainless-steel parallel plate. 

The samples were subjected to periodic torsional shearing between two parallel plates under a strain of 0.1% at a constant normal load of 1 N between a frequency of 0.1 to 100 rad/s at 25 °C in the frequency sweeps. 

Temperature sweeps were carried out with a normal force of 1 N and a constant ramp of 2 °C/min using a constant applied shear strain of 0.1% (γ) and an angular frequency (ω) of 10 rad/s. 

**Figure 1:**
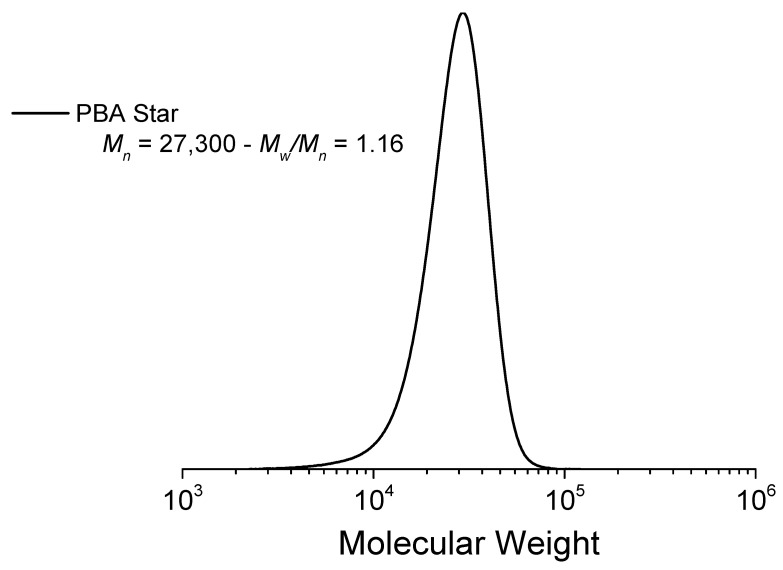
GPC trace of the PBA_4_Br polymer stars. The molecular weight is given relative to linear PMMA standards in THF. The polymer stars were prepared by photoinduced ATRP using a molar ratio of [BA]/[4f-BiB]/[CuBr_2_]:[Me_6_TREN] = 400:1:04:0.24 in 16/64 *v*/*v*% DMF/Anisole. The reaction was conducted under UV light irradiation (λ_max_ = 374 nm at an intensity of 7.2 mW/cm^2^).

Compression tests were conducted in compression mode. The samples were subjected to a normal load increasing linearly with time (loading rate = 0.1 N/s) from 0.08 to 3 N. The load vs. distance curves were converted into the stress σ = F/(π × (D/2)^2^) and compression strain ε = (d_0_ − d)/d_0_, where the initial distance between the plates, d_0_ (corresponding to the initial sample thickness), was determined by the extrapolation to zero load. The modulus was calculated as the slope of the initial linear region of the constructed stress–strain curves, denoted by the red datapoints in Figure 1. 

Stress relaxation tests were conducted at a constant frequency of 10 rad/s and an applied strain of 0.1% at the stated temperature until degradation was complete. Normal force was held at 0.25 N.

### 2.3. Synthesis

#### 2.3.1. Synthesis of Tetrafunctional ATRP Initiator (4f-BiB)

The synthetic protocol for 4f-BiB was modified from a previously reported procedure [43]. Pentaerythritol (2.00 g, 0.0153 mmol) was loaded into a 500 mL round-bottom flask equipped with a magnetic stir bar. Tetrahydrofuran (THF, 100 mL) and pyridine (9.42 mL, 0.0676 mmol) were loaded into the flask. The vessel was sealed with a rubber septum and the contents were sparged by nitrogen gas for 1.5 h. The flask was lowered into an ice bath and α-bromoisobutyryl bromide (11.40 mL, 0.0922 mmol) was added dropwise over 20 min. The reaction was allowed to cool to room temperature overnight. The THF solvent was removed via rotary evaporation to leave a mixture of pyridine salts and crude product deposited on the side of the flask. Ethyl acetate (250 mL) was added to the flask and the contents were vigorously stirred for ten minutes. The suspended solids were removed via gravimetric filtration. The filtrate was washed with 3 *×* 75 mL of 1% HCl (aqueous), 3 × 75 mL sat. NaHCO_3_ (aqueous), and 1 × 50 mL of brine (aqueous). The mixture was dried over MgSO_4_ and filtered through filter paper before excess solvent was removed by rotary evaporation. The crude product was isolated as a white solid. The tetrafunctional initiator was purified via recrystallization from hot methanol. Purity was confirmed by ^1^H NMR (500 MHz, CDCl_3_): δ = 4.33 (s, 8 H); 1.94 (s, 24 H). 

#### 2.3.2. Synthesis of Poly(*n*-butyl acrylate) Star Polymer (PBA_4_-Br) by PhotoATRP

The synthetic protocol used to prepare the PBA star polymer was modified from a previously reported procedure [44]. BA (13.26 g, 104 mmol), 4f-BiB (0.2 g, 0.260 mmol), CuBr_2_ (2.3 mg, 0.01 mmol), Me_6_TREN (0.014 g, 0.062 mmol), DMF (12 mL), and anisole (45 mL) were added to a 100 mL Schlenk flask equipped with a magnetic stir bar. The contents of the flask were degassed by three cycles of freezing in liquid nitrogen, followed by evacuation under vacuum, then thawing to room temperature. The flask was refilled with nitrogen in the final round. An initial aliquot was taken from the reaction mixture for characterization by ^1^H NMR. The photoATRP was started at room temperature by exposing the flask to light of λ_max_ = 374 nm at an intensity of 7.2 mW/cm^2^ overnight [45,46]. Upon completion, the reaction was quenched by turning off the light and exposing the reaction to air. An aliquot of the final reaction mixture was taken for analysis by ^1^H NMR, confirming a conversion of 58% (M_n, th_ = 30,500, DP_sc_ = 58). The crude reaction mixture was diluted with THF and filtered through basic alumina to remove residual catalyst. Extra solvent was removed by rotary evaporation. The sample was allowed to air-dry before analysis by GPC. The stars were isolated as a transparent viscous liquid.

#### 2.3.3. Curing of PBA Networks by RTA-ATRC

An identical procedure was used to cure all polymer networks in this manuscript.

The PBA_4_-Br (0.4 g, 13.1 µmol of polymer stars with a M_n, th_ = 30,500, 1 eq.) was added to 1-dram vials as a 50 vol% stock solution in THF (0.4 mL solvent, or 0.8 mL mixture). PMDETA (14 µL, 66 µmol, 5 eq.), nitrosobenzene (0.0056 g, 52.5 µmol, 4 eq.), and CuBr_2_ (0.003 g, 13.1 µmol, 1 eq.) were mixed into the vial. The vials were sealed with a rubber septum and electrical tape. The mixtures were degassed by nitrogen sparging for 8 min. Then, Sn(EH)_2_ (0.042 mL, 130 µmol, 10 eq.) was added to the mixtures via a purged syringe. The contents of the vials were shaken to ensure the mixture was homogeneous prior to curing overnight at 60 °C. The resulting disc-shaped gels were then removed from the vials. The gels were isolated as a soft orange solid. The gels were purified by swelling against DMF (200 mL) to remove unreacted components. The solvent was exchanged twice a day, for a total of 3 days. The gels were dried in an oven (40 °C) under vacuum to remove the solvent. The dry weight (W_d_) of the gels was between 0.19 and 0.26 g, corresponding to a 47–67% gel fraction. 

## 3. Results

### 3.1. Network Synthesis

**Scheme 2:**
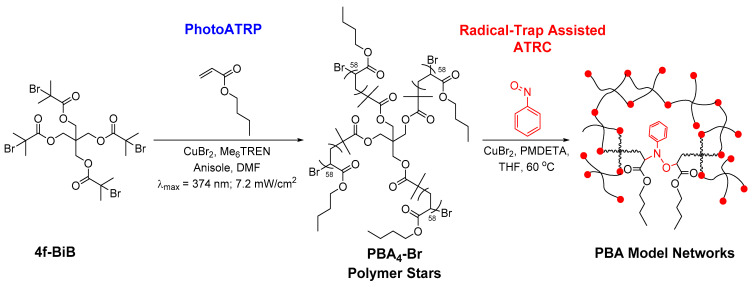
Synthetic route used to prepare degradable PBA model networks. PBA stars were prepared by grafting-from photoATRP using a tetrafunctional initiator as the core. The polymer stars were cured into crosslinked networks by RTA-ATRC with the nitrsobenzene radical trap.

Four-arm polymer stars were prepared by photoATRP of BA from a tetrafunctional α-bromoisobutyrate core (4f-BiB) using the grafting-from approach (Scheme 2) [43,47,48,49]. PhotoATRP enables the synthesis of polyacrylates and methacrylates using ppm loadings of copper catalyst by continuous regeneration of the CuBr/L activator by reduction of the CuBr_2_/Me_6_TREN deactivator with excess tertiary amines as the reducing agent [43,47,48,49]. PhotoATRP can be conducted in the presence of photoredox catalysts, such as quantum dots or other photosensitizers [50,51], as well as without transition metals in a metal-free ATRP [52,53,54]. The polymerization proceeded under UV irradiation with a CuBr_2_/Me_6_TREN catalyst with excess Me_6_TREN as the reducing agent [55]. The polymerization used a molar ratio [BA]/[4f-BiB]/[CuBr_2_]:[Me_6_TREN] = 400:1:04:0.24 in 16/64 *v*/*v*% DMF/Anisole. The reaction reached 58% conversion after stirring overnight under UV light (λ_max_ = 374 nm at an intensity of 7.2 mW/cm^2^) irradiation, providing 4-arm polymer stars with a theoretical 58 repeating units of BA per arm. The GPC of the purified polymer stars had a M_n, GPC_ = 27,300, comparable to the theoretical M_n, th_ = 30,500. The sample had a narrow molecular weight distribution (Ð = 1.16) and did not have high molecular weight shouldering visible in the GPC trace (Figure 1). 

The high retention of end-group functionality of polymers prepared by ATRP [56] enabled curing of polymer stars into crosslinked elastomers via radical termination upon activation by an activators generated by electron transfer (AGET) [57] mechanism in the presence of a nitrosobenzene radical trap and a large excess of Sn(EH)_2_ reducing agent [58]. The curing recipe utilized a molar ratio of [NBz]/[PBA_4_Br]/[CuBr_2_]/[PMDETA]/[Sn(EH)_2_] = 4/1/1/5/10 at 60 °C, which provided polymer networks crosslinked with thermally labile alkoxyamine linkages (Scheme 2). The gel fractions of the networks cured using the discussed approach were between 47% and 67%, as determined by gravimetry in the dry state after removal of sol by swelling gels in DMF solvent.

### 3.2. Network Characterization

#### 3.2.1. Mechanical Properties of the Networks

**Figure 2:**
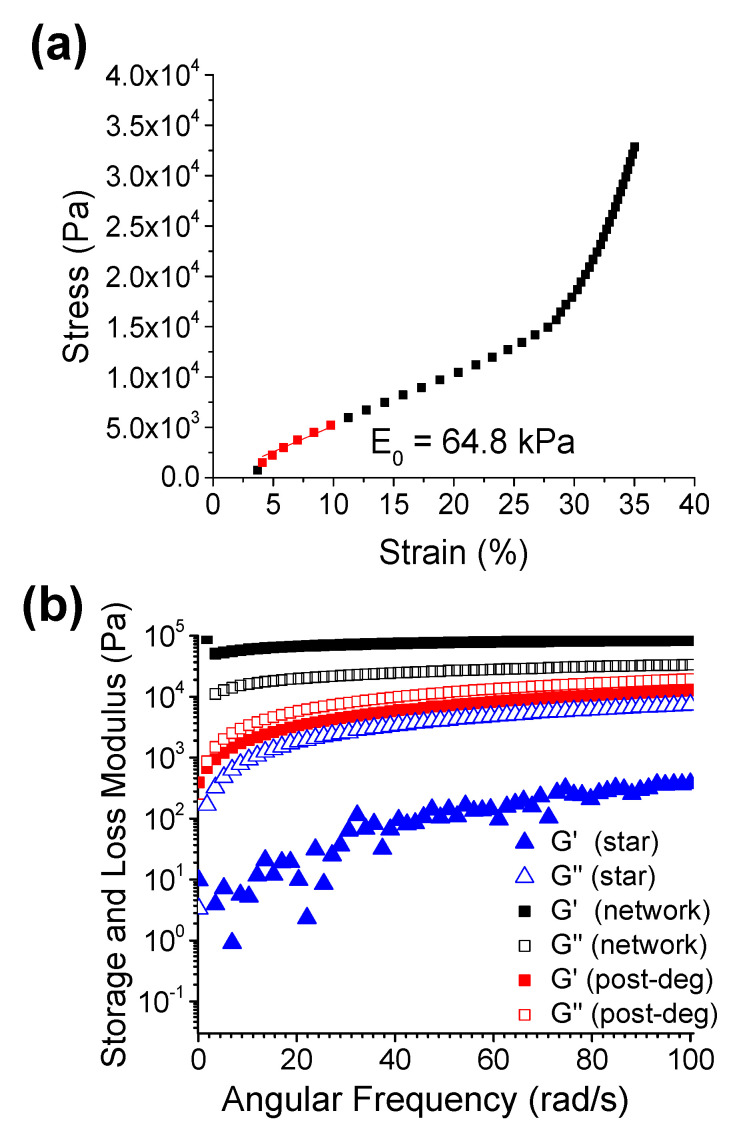
(**a**) Compression test of PBA elastomer at room temperature. (**b**) Frequency sweep of the polymer stars (blue), crosslinked network (black), and degraded network recast as a film (red) from a range of 0.1 to 100 rad/s with a 1 N normal force and T = 25 °C.

The modulus of the cured polymer network was 64.8 kPa, as determined by a compression test at ambient temperature (Figure 2a). The successful crosslinking reaction was confirmed by frequency sweep across a frequency of 0.01–100 rad/s at an applied strain of 1 N (Figure 2b). The storage modulus (G′) values were greater than the loss modulus (G″) across the range of frequencies, confirming a solid but rubbery character of the crosslinked elastomer. In contrast, the original PBA stars behaved as a soft, viscous liquid because PBA is significantly above its glass transition temperature (T_g_ = −55 °C) at room temperature.

**Figure 3:**
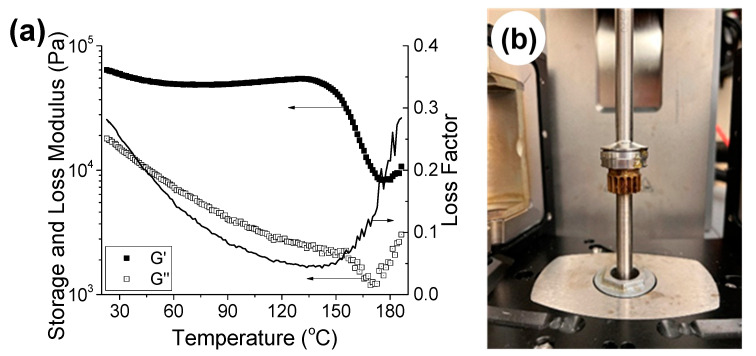
(**a**) Temperature sweep of the PBA elastomer from a temperature of 25 to 190 °C. The loss factor is the tan δ = G″/G′. The sweep was conducted at a frequency of 10 rad/s and a constant applied shear strain of 0.1%. The applied normal force was 1 N. (**b**) Picture of the DMA set-up after network degradation during the temperature sweep experiment. The degraded network became a liquid which flowed over the side of the sample plate.

Figure 3a shows the temperature dependence of the storage and loss moduli overlayed with the loss factor (i.e., tan δ = G″/G′) from room temperature up to 190 °C. The temperature sweep shows that the network structure was maintained across a long rubbery plateau between 25 and 135 °C. Degradation of the network was observed above 135 °C as a concurrent drop in the storage modulus by an order of magnitude, and a drop in loss modulus by a factor of two. This led to a spike in the loss factor corresponding to a loss of mechanical integrity in the network. It should be noted that degradation of the network led to a solid to liquid transition which increased the sample diameter, that could lead to some inaccuracies in rheological characterization during sample degradation (Figure 3b). The degraded network was soluble in THF.

**Figure 4:**
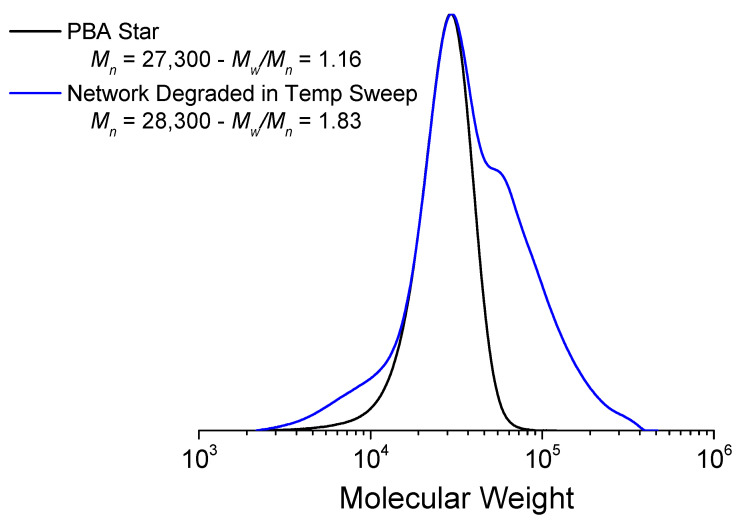
GPC trace of the degraded network after the temperature sweep overlayed with the trace of the PBA_4_Br star polymer precursor. The molecular weight is relative to linear PMMA standards in THF.

A frequency sweep of the degraded product re-casted as a film showed a liquid character, indicating successful cleavage of the majority of alkoxyamine crosslinks, however the modulus was still higher than that of the original PBA stars (Figure 2b). GPC of the degraded network is shown in Figure 4. The degraded polymer had a M_n, GPC_ = 28,300 (Ð = 1.83) comparable to the original 4-arm star polymer, with an additional high molecular weight shoulder characteristic of polyacrylates terminated by coupling [59,60]. There was a lower molecular weight shoulder with a M_n, GPC_ ~ 7000. This may be attributed to cleavage of some star-arms from the core at a high temperature under shear, as the molecular weight of the impurity closely matches the theoretical molecular weight of one PBA arm (i.e., M_n, th, arm_ = 7400).

**Figure 5:**
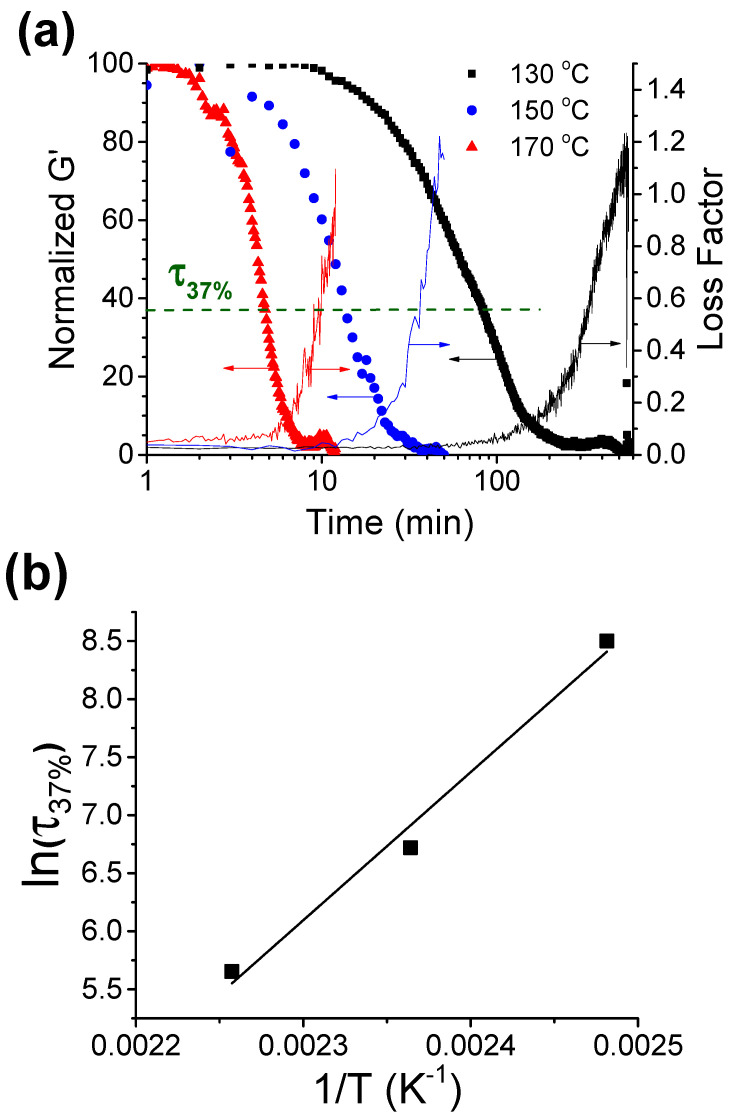
(**a**) Stress relaxation of crosslinked PBA networks. Samples were held at a frequency of 10 rad/s and an applied strain of 0.1% at a given temperature until degradation was complete. The loss factor is the tan δ = G″/G′. (**b**) Arrhenius plot of the logarithm of chain relaxation at 37% G_0_ (ln τ_37%_) against inverse temperature for stress relaxation experiments.

Stress relaxation experiments were performed at 130, 150, and 170 °C using polymer networks cured using an identical recipe to characterize the rate of alkoxyamine decomposition at an applied strain of 0.1% (Figure 5a). Similar to the temperature sweep experiment, polymer networks exposed to elevated temperatures at the same applied strain were observed to degrade by a concurrent decrease in storage moduli and an increase in loss factor after sample degradation. As expected, the rate of sample degradation increased with the relaxation temperature, in agreement with the increase in the rate of alkoxyamine decomposition observed in other reports. Networks degraded after nearly 2 h at 130 °C. The decomposition was shortened to 20 min at 150 °C, and ~7 min at 170 °C. A quantitative measurement of the temperature-dependent viscous flow of a network with exchangeable crosslinks was determined by an Arrhenius plot of the characteristic relaxation time (τ_37%_) vs. temperature. The τ_37%_ is defined as the time when G′ = 0.37G′_0_. The Arrhenius plot was linear for the polymer networks over the tested temperature range, providing an E_a_ = 106.0 ± 12.4 kJ/mol (Figure 5b), a value comparable to the reported E_a_ of thermal cleavage of typical alkoxyamines [61].

#### 3.2.2. Degradation in Solution

**Figure 6:**
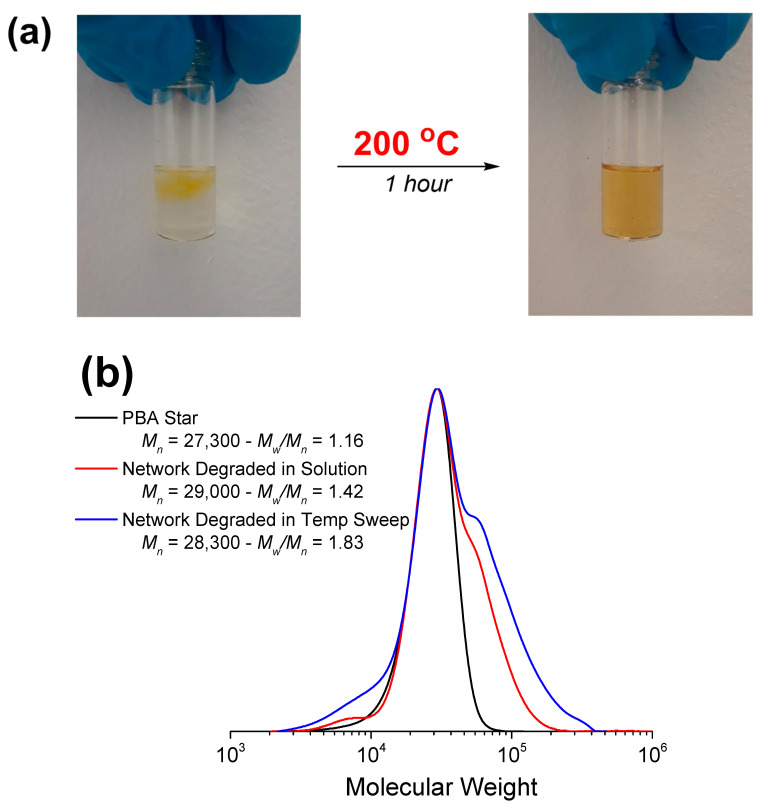
(**a**) The crosslinked network was swollen in 1,2,4-trichlorobenzene (40 mg sample/2 mL solvent) and heated at 200 °C for 60 min. (**b**) The GPC of PBA star polymer precursor and soluble components after degradation.

The degradability of the network was assessed under controlled conditions. The crosslinked network was swollen in 1,2,4-trichlorobenzene (40 mg sample/2 mL solvent) and heated at 200 °C for 60 min to ensure degradation of the majority of alkoxyamine crosslinks. The network was fully soluble in the solvent after heating, indicating that most crosslinks were cleaved after thermal treatment (Figure 6a). The GPC trace of the degraded network was comparable to the original star polymer. In line with the network degraded during the temperature sweep, a fraction of coupled polymer impurity was present in this sample (Figure 6b). This indicates that most polymer stars were cured with alkoxyamine crosslinks, and a fraction of stars were coupled by standard biradical combination without the radical trap. The fraction of cleaved single arms at M_n, GPC_ ~ 7000 was lower than in the sample degraded in the temperature sweep.

#### 3.2.3. Thermogravimetric Analysis

**Figure 7:**
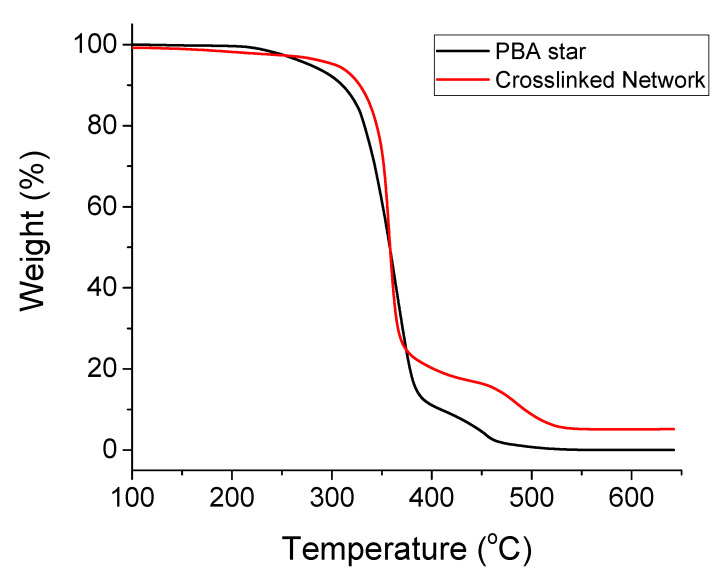
TGA of the star polymer and crosslinked network.

The TGA traces of the network in Figure 7 showed that the network had T_d,10%_ = 328 °C, comparable to the T_d,10%_ = 310 °C of the PBA star precursor, which further supported degradation of alkoxyamines as the route of primary degradation. The crosslinked network had a higher tendency toward char formation, which may be attributed to differences in end-group functionality [62].

## 4. Conclusions

Poly(*n*-butyl acrylate) polymer stars with well-defined topology were prepared by photoinduced ATRP. The stars were used as multi-functional macromonomers in the synthesis of soft model networks via RTA-ATRC with a nitrosobenzene radical trap. The crosslinked networks exhibited a low modulus and were degraded above 130 °C in the solid state. The temperature sweep data showed that the network retained a consistent rubbery plateau prior to degradation. The dissolution of the solid network to liquid stars was observed as an abrupt decrease in both storage and loss moduli after network integrity was compromised. The degradation rate of the alkoxyamine-crosslinked network was accelerated at higher temperatures. GPC of the degraded polymers showed that the inversion of model network topology was achievable both in solution and in the solid state. The high molecular weight shouldering in the GPC traces of degraded polymers indicated bimolecular coupling of stars via radical termination.

## Data Availability

Data are contained within the article.

## References

[1] Gu Y., Zhao J., Johnson J.A. (2019). A (Macro) Molecular-Level Understanding of Polymer Network Topology. Trends Chem..

[2] Daniel W.F., Burdyńska J., Vatankhah-Varnoosfaderani M., Matyjaszewski K., Paturej J., Rubinstein M., Dobrynin A.V., Sheiko S.S. (2016). Solvent-free, supersoft and superelastic bottlebrush melts and networks. Nat. Mater..

[3] Reynolds V.G., Mukherjee S., Xie R., Levi A.E., Atassi A., Uchiyama T., Wang H., Chabinyc M.L., Bates C.M. (2020). Super-soft solvent-free bottlebrush elastomers for touch sensing. Mater. Horiz..

[4] Liang H., Sheiko S.S., Dobrynin A.V. (2018). Supersoft and hyperelastic polymer networks with brushlike strands. Macromolecules.

[5] Cuthbert J., Balazs A.C., Kowalewski T., Matyjaszewski K. (2020). STEM gels by controlled radical polymerization. Trends Chem..

[6] Pandey P., Chauhan R. (2001). Membranes for gas separation. Prog. Polym. Sci..

[7] Javaid A. (2005). Membranes for solubility-based gas separation applications. Chem. Eng. J..

[8] Sun Q., Dai Z., Meng X., Xiao F.-S. (2015). Porous polymer catalysts with hierarchical structures. Chem. Soc. Rev..

[9] Huang J., Turner S.R. (2018). Hypercrosslinked polymers: A review. Polym. Rev..

[10] Rikkou M.D., Kolokasi M., Matyjaszewski K., Patrickios C.S. (2010). End-linked amphiphilic polymer conetworks: Synthesis by sequential atom transfer radical polymerization and swelling characterization. J. Polym. Sci. Part A Polym. Chem..

[11] Patrickios C.S., Matyjaszewski K. (2021). Amphiphilic polymer co-networks: 32 years old and growing stronge—A perspective. Polym. Int..

[12] Cuthbert J., Wanasinghe S.V., Matyjaszewski K., Konkolewicz D. (2021). Are RAFT and ATRP Universally Interchangeable Polymerization Methods in Network Formation?. Macromolecules.

[13] Rikkou-Kalourkoti M., Loizou E., Porcar L., Matyjaszewski K., Patrickios C.S. (2012). End-linked, amphiphilic, degradable polymer conetworks: Synthesis by sequential atom transfer radical polymerization using a bifunctional, cleavable initiator. Polym. Chem..

[14] Yoon J.A., Kamada J., Koynov K., Mohin J., Nicolaÿ R., Zhang Y., Balazs A.C., Kowalewski T., Matyjaszewski K. (2012). Self-Healing Polymer Films Based on Thiol–Disulfide Exchange Reactions and Self-Healing Kinetics Measured Using Atomic Force Microscopy. Macromolecules.

[15] Kamada J., Koynov K., Corten C., Juhari A., Yoon J.A., Urban M.W., Balazs A.C., Matyjaszewski K. (2010). Redox responsive behavior of thiol/disulfide-functionalized star polymers synthesized via atom transfer radical polymerization. Macromolecules.

[16] Kolmakov G.V., Matyjaszewski K., Balazs A.C. (2009). Harnessing labile bonds between nanogel particles to create self-healing materials. ACS Nano.

[17] Self J.L., Sample C.S., Levi A.E., Li K., Xie R., de Alaniz J.R., Bates C.M. (2020). Dynamic bottlebrush polymer networks: Self-healing in super-soft materials. J. Am. Chem. Soc..

[18] Mark J., Sullivan J. (1977). Model networks of end-linked polydimethylsiloxane chains. I. Comparisons between experimental and theoretical values of the elastic modulus and the equilibrium degree of swelling. J. Chem. Phys..

[19] Villar M.A., Vallés E.M. (1995). Influence of the final extent of reaction on the structure of model polydimethylsiloxane networks obtained by the end-linking hydrosilation reaction. Polym. Bull..

[20] Johnson J.A., Lewis D.R., Díaz D.D., Finn M., Koberstein J.T., Turro N.J. (2006). Synthesis of degradable model networks via ATRP and click chemistry. J. Am. Chem. Soc..

[21] Johnson J.A., Finn M.G., Koberstein J.T., Turro N.J. (2007). Synthesis of Photocleavable Linear Macromonomers by ATRP and Star Macromonomers by a Tandem ATRP–Click Reaction:  Precursors to Photodegradable Model Networks. Macromolecules.

[22] Shih H., Lin C.-C. (2012). Cross-linking and degradation of step-growth hydrogels formed by thiol–ene photoclick chemistry. Biomacromolecules.

[23] Cok A.M., Zhou H., Johnson J.A. (2013). Synthesis of Model Network Hydrogels via Tetrazine-O lefin Inverse Electron Demand Diels-A lder Cycloaddition. Macromol. Symp..

[24] Jia Y., Matt Y., An Q., Wessely I., Mutlu H., Theato P., Bräse S., Llevot A., Tsotsalas M. (2020). Dynamic covalent polymer networks via combined nitroxide exchange reaction and nitroxide mediated polymerization. Polym. Chem..

[25] Wang G., Huang J. (2014). Versatility of radical coupling in construction of topological polymers. Polym. Chem..

[26] Sarbu T., Lin K.-Y., Spanswick J., Gil R.R., Siegwart D.J., Matyjaszewski K. (2004). Synthesis of hydroxy-telechelic poly (methyl acrylate) and polystyrene by atom transfer radical coupling. Macromolecules.

[27] Sarbu T., Lin K.-Y., Ell J., Siegwart D.J., Spanswick J., Matyjaszewski K. (2004). Polystyrene with Designed Molecular Weight Distribution by Atom Transfer Radical Coupling. Macromolecules.

[28] Wang S., Zhang K., Chen Y., Xi F. (2014). Isomeric Dicyclic Polymers via Atom Transfer Radical Polymerization and Atom Transfer Radical Coupling Cyclization. Macromolecules.

[29] Luo X., Wang G., Huang J. (2009). Preparation of H-shaped ABCAB terpolymers by atom transfer radical coupling. J. Polym. Sci. Part A Polym. Chem..

[30] Nicolaÿ R., Marx L., Hémery P., Matyjaszewski K. (2007). Synthesis of multisegmented degradable polymers by atom transfer radical cross-coupling. Macromolecules.

[31] Li I., Howell B.A., Matyjaszewski K., Shigemoto T., Smith P.B., Priddy D.B. (1995). Kinetics of decomposition of 2,2,6,6-tetramethyl-1-(1-phenylethoxy)piperidine and its implications on nitroxyl-mediated styrene polymerization. Macromolecules.

[32] Voter A.F., Tillman E.S., Findeis P.M., Radzinski S.C. (2012). Synthesis of macrocyclic polymers formed via intramolecular radical trap-assisted atom transfer radical coupling. ACS Macro Lett..

[33] Valente C.J., Schellenberger A.M., Tillman E.S. (2014). Dimerization of poly (methyl methacrylate) chains using radical trap-assisted atom transfer radical coupling. Macromolecules.

[34] Arce M.M., Pan C.W., Thursby M.M., Wu J.P., Carnicom E.M., Tillman E.S. (2016). Influence of solvent on radical trap-assisted dimerization and cyclization of polystyrene radicals. Macromolecules.

[35] Butcher W.E., Radzinski S.C., Tillman E.S. (2013). Selective formation of diblock copolymers using radical trap-assisted atom transfer radical coupling. J. Polym. Sci. Part A Polym. Chem..

[36] Blackburn S.C., Myers K.D., Tillman E.S. (2015). Macrocyclic poly (methyl acrylate) and macrocyclic poly (methyl acrylate-block-styrene) synthesized by radical trap-assisted atom transfer radical coupling. Polymer.

[37] McFadden B.D., Arce M.M., Carnicom E.M., Herman J., Abrusezze J., Tillman E.S. (2016). Radical Trap-Assisted Atom Transfer Radical Coupling of Diblock Copolymers as a Method of Forming Triblock Copolymers. Macromol. Chem. Phys..

[38] Andry J.J., Lee J.J., Wu J., Xia K., Tillman E.S. (2021). Universal chain-end coupling conditions for brominated polystyrenes, polyacrylates, and polymethacrylates. Processes.

[39] Zhang Z., Wang G., Huang J. (2011). Synthesis of H-shaped A3BA3 copolymer by methyl-2-nitrosopropane induced single electron transfer nitroxide radical coupling. J. Polym. Sci. Part A Polym. Chem..

[40] Telitel S., Amamoto Y., Poly J., Morlet-Savary F., Soppera O., Lalevee J., Matyjaszewski K. (2014). Introduction of self-healing properties into covalent polymer networks via the photodissociation of alkoxyamine junctions. Polym. Chem..

[41] Su J., Amamoto Y., Nishihara M., Takahara A., Otsuka H. (2011). Reversible cross-linking of hydrophilic dynamic covalent polymers with radically exchangeable alkoxyamines in aqueous media. Polym. Chem..

[42] Li L., Chen X., Jin K., Rusayyis M.B., Torkelson J.M. (2021). Arresting Elevated-Temperature Creep and Achieving Full Cross-Link Density Recovery in Reprocessable Polymer Networks and Network Composites via Nitroxide-Mediated Dynamic Chemistry. Macromolecules.

[43] van Ravensteijn B.G.P., Bou Zerdan R., Helgeson M.E., Hawker C.J. (2019). Minimizing Star–Star Coupling in Cu(0)-Mediated Controlled Radical Polymerizations. Macromolecules.

[44] Martinez M.R., Sobieski J., Lorandi F., Fantin M., Dadashi-Silab S., Xie G., Olszewski M., Pan X., Ribelli T.G., Matyjaszewski K. (2020). Understanding the Relationship between Catalytic Activity and Termination in photoATRP: Synthesis of Linear and Bottlebrush Polyacrylates. Macromolecules.

[45] Pan X., Tasdelen M.A., Laun J., Junkers T., Yagci Y., Matyjaszewski K. (2016). Photomediated controlled radical polymerization. Prog. Polym. Sci..

[46] Konkolewicz D., Schröder K., Buback J., Bernhard S., Matyjaszewski K. (2012). Visible light and sunlight photoinduced ATRP with ppm of Cu catalyst. ACS Macro Lett..

[47] Cuthbert J., Yerneni S.S., Sun M., Fu T., Matyjaszewski K. (2019). Degradable polymer stars based on tannic acid cores by ATRP. Polymers.

[48] Matyjaszewski K., Miller P.J., Pyun J., Kickelbick G., Diamanti S. (1999). Synthesis and Characterization of Star Polymers with Varying Arm Number, Length, and Composition from Organic and Hybrid Inorganic/Organic Multifunctional Initiators. Macromolecules.

[49] Miller P.J., Matyjaszewski K. (1999). Atom transfer radical polymerization of (meth) acrylates from poly (dimethylsiloxane) macroinitiators. Macromolecules.

[50] Zhu Y., Egap E. (2021). Light-Mediated Polymerization Induced by Semiconducting Nanomaterials: State-of-the-Art and Future Perspectives. ACS Polym. Au.

[51] Zhu Y., Jin T., Lian T., Egap E. (2021). Enhancing the efficiency of semiconducting quantum dot photocatalyzed atom transfer radical polymerization by ligand shell engineering. J. Chem. Phys..

[52] Wang J., Yuan L., Wang Z., Rahman M.A., Huang Y., Zhu T., Wang R., Cheng J., Wang C., Chu F. (2016). Photoinduced Metal-Free Atom Transfer Radical Polymerization of Biomass-Based Monomers. Macromolecules.

[53] Theriot J.C., McCarthy B.G., Lim C.-H., Miyake G.M. (2017). Organocatalyzed Atom Transfer Radical Polymerization: Perspectives on Catalyst Design and Performance. Macromol. Rapid Commun..

[54] Discekici E.H., Anastasaki A., Read de Alaniz J., Hawker C.J. (2018). Evolution and Future Directions of Metal-Free Atom Transfer Radical Polymerization. Macromolecules.

[55] Ribelli T.G., Konkolewicz D., Bernhard S., Matyjaszewski K. (2014). How are Radicals (Re)Generated in Photochemical ATRP?. J. Am. Chem. Soc..

[56] Anastasaki A., Willenbacher J., Fleischmann C., Gutekunst W.R., Hawker C.J. (2017). End group modification of poly (acrylates) obtained via ATRP: A user guide. Polym. Chem..

[57] Min K., Gao H., Matyjaszewski K. (2005). Preparation of homopolymers and block copolymers in miniemulsion by ATRP using activators generated by electron transfer (AGET). J. Am. Chem. Soc..

[58] Domingues K.M., Tillman E.S. (2010). Radical–radical coupling of polystyrene chains using AGET ATRC. J. Polym. Sci. Part A Polym. Chem..

[59] Ribelli T.G., Augustine K.F., Fantin M., Krys P., Poli R., Matyjaszewski K. (2017). Disproportionation or Combination? The Termination of Acrylate Radicals in ATRP. Macromolecules.

[60] Xie G., Martinez M.R., Daniel W.F.M., Keith A.N., Ribelli T.G., Fantin M., Sheiko S.S., Matyjaszewski K. (2018). Benefits of Catalyzed Radical Termination: High-Yield Synthesis of Polyacrylate Molecular Bottlebrushes without Gelation. Macromolecules.

[61] Audran G., Blyth M.T., Coote M.L., Gescheidt G., Hardy M., Havot J., Holzritter M., Jacoutot S., Joly J.-P., Marque S.R. (2021). Homolysis/mesolysis of alkoxyamines activated by chemical oxidation and photochemical-triggered radical reactions at room temperature. Org. Chem. Front..

[62] Yu-Hsiang H., Chen C., Wang C. (2004). Thermal degradation kinetics of poly (*n*-butyl acrylate) initiated by lactams and thiols. Polym. Degrad. Stab..

